# Dual-Outlet Stomach-Partitioning Gastrojejunostomy for Malignant Duodenal Obstruction: A Novel Palliative Bypass Technique

**DOI:** 10.70352/scrj.cr.25-0483

**Published:** 2025-10-15

**Authors:** Kensuke Kudou, Eiji Oki, Tetsuro Kawazoe, Sho Nambara, Yasuo Tsuda, Tomonori Nakanoko, Koji Ando, Tomoharu Yoshizumi

**Affiliations:** 1 Department of Surgery and Science, Graduate School of Medical Sciences, Kyushu University, Fukuoka, Fukuoka, Japan; 2 Department of Gastroenterological Surgery, Cancer Institute Hospital of Japanese Foundation for Cancer Research, Tokyo, Japan

**Keywords:** gastrojejunostomy, stomach-partitioning, duodenal obstruction, gastrointestinal bypass, palliative surgery

## Abstract

**INTRODUCTION:**

Stomach-partitioning gastrojejunostomy (SPGJ) is widely performed for malignant gastric outlet obstruction; however, its utility may be limited when the obstruction is located in the distal duodenum, where digestive fluid stasis can become problematic. We devised a novel modification, termed dual-outlet stomach-partitioning gastrojejunostomy (DO-SPGJ), to address this limitation by adding a 2nd gastrojejunostomy distal to the gastric partition.

**CASE PRESENTATION:**

A 67-year-old man with a tumor in the 3rd portion of the duodenum was diagnosed with squamous cell carcinoma without distant metastasis. The tumor was deemed unresectable due to invasion of the superior mesenteric artery. After 2 months of systemic chemotherapy, the patient developed symptoms of gastric outlet obstruction. A laparoscopic modified SPGJ was performed, involving a standard proximal gastrojejunostomy and an additional distal anastomosis between the gastric antrum and jejunum. The postoperative course was uneventful, and oral intake was successfully resumed.

**CONCLUSIONS:**

This dual-anastomosis approach allows for both food bypass and drainage of digestive secretions, addressing the limitation of conventional SPGJ in cases of distal duodenal obstruction. The technique may also mitigate complications related to fluid stagnation, such as cholangitis or pancreatitis. This novel technique may represent a viable surgical option for select patients with unresectable malignant obstruction of the distal duodenum, especially when fluid stasis is a concern.

## Abbreviations

DO-SPGJ
: dual-outlet stomach-partitioning gastrojejunostomy
LSPGJ
: laparoscopic stomach-partitioning gastrojejunostomy
SPGJ
: stomach-partitioning gastrojejunostomy

## INTRODUCTION

Malignant tumors and severe inflammation affecting the stomach, duodenum, or pancreas can cause irreversible obstruction of the duodenum or jejunum. When such obstruction becomes pronounced, patients may suffer from vomiting, abdominal pain, and severely impaired quality of life. In cases of advanced gastric outlet obstruction, some form of intervention is required to decompress the dilated proximal stomach and to restore alimentary passage.

While resection of the causative lesion is ideal, surgical access to the duodenum and proximal jejunum is technically challenging due to the proximity of major surrounding organs, often making resection unfeasible. For unresectable obstructions, gastrojejunostomy or endoscopic stenting is commonly selected. Although endoscopic stenting is minimally invasive, it carries the risks of stent migration, restenosis, and perforation. Gastrojejunostomy is often more reliable in restoring passage.

In 1881, the 1st gastrojejunostomy for unresectable pyloric gastric cancer was performed by Wölfler, a student of Billroth, the same year Billroth performed the 1st gastrectomy for gastric cancer.^1)^ The initial procedure involved a Billroth II reconstruction with Braun anastomosis and remained widely used for decades. However, approximately half of the cases experienced delayed gastric emptying, resulting in inadequate bypass function and impaired oral intake.

To address this issue, Devine proposed a modification in 1925, involving complete division of the stomach between the stenotic and healthy segments, with the jejunum anastomosed to the proximal healthy segment.^2)^ However, this method raised concerns of gastric content stagnation and potential “blow-out” in the distal remnant stomach. To prevent this, Kajitani et al.^3)^ reported in 1971 a modified approach, in which a small tunnel (approximately 2 cm in diameter) was preserved on the lesser curvature, creating an incomplete gastric partition. This prevented food inflow into the stenotic region while allowing drainage from the distal stomach, minimizing the risk of blow-out. Furthermore, this approach allows for endoscopic access to the distal stomach postoperatively, facilitating additional interventions.

This technique, referred to as the modified Devine procedure or SPGJ, gained popularity. With the advent of stapling devices and laparoscopic surgery in the 1990s, LSPGJ has become the standard surgical approach.^4,5)^

Although conventional gastrojejunostomy and duodenojejunostomy are widely used for palliative bypass, they may be suboptimal in cases involving distal duodenal obstruction. In standard gastrojejunostomy without stomach partitioning, food may still enter the obstructed duodenum, causing persistent symptoms. Duodenojejunostomy, while anatomically direct, can be technically challenging when the obstruction is located in the 3rd or 4th portion of the duodenum, especially when there is tumor invasion near the major duodenal papilla or superior mesenteric vessels. SPGJ can effectively prevent food entry into the distal stomach and duodenum; however, in cases of obstruction located in the far distal duodenum—as in the present case—there remains concern regarding the stagnation of bile and pancreatic juice within the distal stomach and duodenum due to impaired outflow.

To resolve this problem, we devised a novel modification of SPGJ, termed DO-SPGJ. In addition to conventional gastrojejunostomy on the proximal side of the incomplete partition, we added a 2nd gastrojejunostomy on the distal side to create an outflow tract for refluxed digestive fluids.

## CASE PRESENTATION

A 67-year-old man with a history of laparoscopic low anterior resection and ileostomy for rectal cancer, subsequent ileostomy closure, cholecystectomy, and percutaneous coronary intervention for myocardial infarction 1 year earlier, presented with postprandial back pain. Imaging at a previous hospital revealed a mass in the 3rd portion of the duodenum, and he was referred to our institution, Kyushu University.

**Fig. 1:**
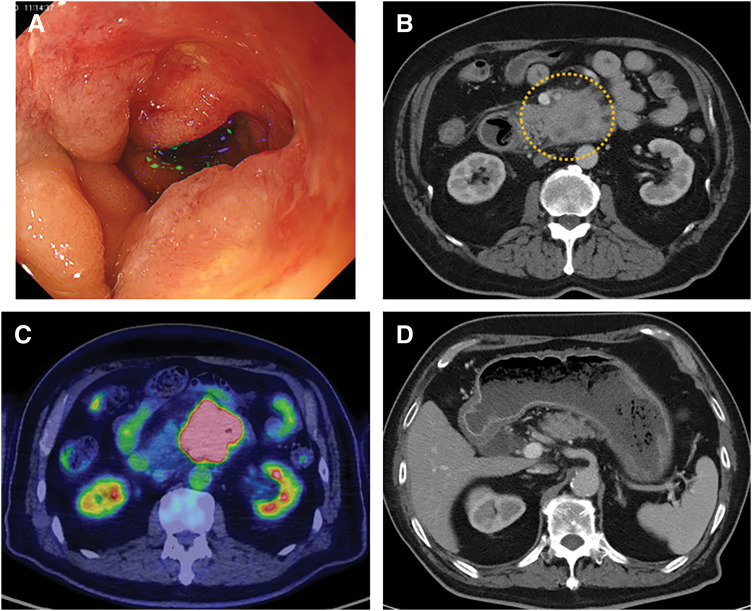
Preoperative imaging and endoscopic findings. (**A**) Upper gastrointestinal endoscopy revealed a circumferential, irregular ulcerative lesion in the 3rd portion of the duodenum, causing luminal narrowing. (**B**) Contrast-enhanced CT demonstrated an approximately 50-mm, irregularly marginated mass in the 3rd portion of the duodenum. (**C**) PET-CT showed increased fluorodeoxyglucose uptake in the 3rd portion of the duodenum, consistent with the tumor site. (**D**) Axial CT showed dilation of the stomach and proximal duodenum due to obstruction.

Further diagnostic work-up, including esophagogastroduodenoscopy, contrast-enhanced CT, PET-CT, and histopathological examination of biopsy specimens, revealed advanced squamous cell carcinoma of the 3rd portion of the duodenum. Upper gastrointestinal endoscopy revealed a circumferential, irregular ulcerative lesion in the 3rd portion of the duodenum, causing luminal narrowing (**Fig. 1A**). CT revealed an ill-defined mass, measuring approximately 50 mm in maximum diameter, in the 3rd portion of the duodenum (**Fig. 1B**). PET-CT showed increased fluorodeoxyglucose uptake in the 3rd portion of the duodenum, corresponding to the tumor site (**Fig. 1C**). PET-CT showed no evidence of distant metastasis. However, the tumor was judged unresectable due to invasion of the superior mesenteric artery, and systemic chemotherapy with FOLFOX plus nivolumab was initiated.

On day 11 of the 1st cycle, the patient passed melena and received a transfusion of 4 units of red blood cells. Anemia subsequently improved, and chemotherapy was continued. Two months later, he developed abdominal pain, distension, and diarrhea. CT demonstrated marked dilation of the stomach and duodenum proximal to the tumor, consistent with duodenal obstruction (**Fig. 1D**). Gastrojejunostomy was indicated.

### Surgical procedure

Surgery was performed 76 days after chemotherapy initiation. After lysing adhesions from previous surgeries, laparoscopic access was obtained using 5 ports. DO-SPGJ was planned to simultaneously achieve alimentary bypass and drainage of digestive secretions.

**Fig. 2:**
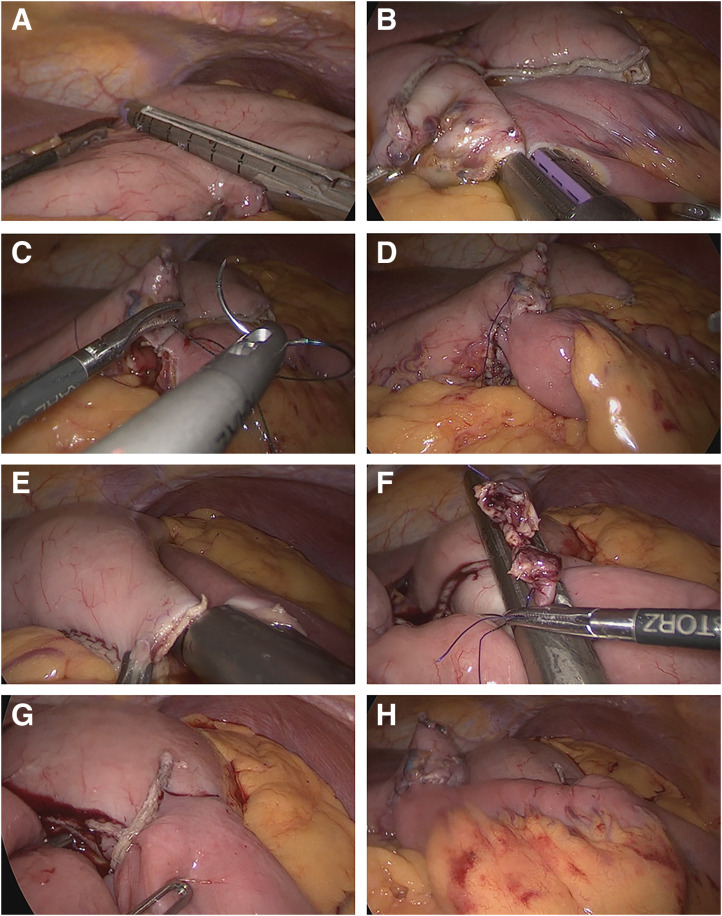
Intraoperative procedures of DO-SPGJ. (**A**) A stapled, incomplete gastric partition was created at the lower gastric body, preserving a 2-cm tunnel at the lesser curvature. (**B**) A side-to-side, antiperistaltic gastrojejunostomy was created between the gastric antrum and a jejunal loop 20 cm distal to the ligament of Treitz. (**C**) The barbed suture is being placed to close the common enterotomy, which was located on the posterior gastric wall and difficult to access with a stapler. (**D**) Completed closure of the common enterotomy with a continuous barbed suture. (**E**) A second, side-to-side gastrojejunostomy was performed between the gastric body proximal to the partition and a jejunal segment located 10 cm distal to the 1st anastomosis. (**F**) Three stay sutures were placed to temporarily close the common enterotomy. (**G**) The enterotomy was closed using a linear stapler. (**H**) Intraoperative view after completion of both gastrojejunostomies. DO-SPGJ, dual-outlet stomach-partitioning gastrojejunostomy

The greater omentum was dissected along the greater curvature starting from the angular incisure toward the gastric body to a level suitable for safe anastomosis. A stapled, incomplete gastric partition was created at the lower gastric body, leaving a 2-cm tunnel on the lesser curvature (**Fig. 2A**).

Due to the obstruction in the 3rd portion of the duodenum, we added an additional gastrojejunostomy between the gastric antrum distal to the partition and a jejunal loop approximately 20 cm distal to the ligament of Treitz. A side-to-side, antiperistaltic anastomosis was created using a linear stapler. For the distal gastrojejunostomy, a 60-mm linear stapler was used. The cartridge was partially inserted, resulting in a side-to-side anastomosis of approximately 5 cm. This relatively short anastomosis was intentionally constructed, as its primary purpose was to drain refluxed bile and pancreatic juice, rather than to allow food passage (**Fig. 2B**). As the common enterotomy site was located on the posterior wall of the stomach, it was technically difficult to close it with a stapler due to the limited angle. Therefore, the enterotomy was closed with a continuous barbed suture (**Fig. 2C** and **2D**).

**Fig. 3:**
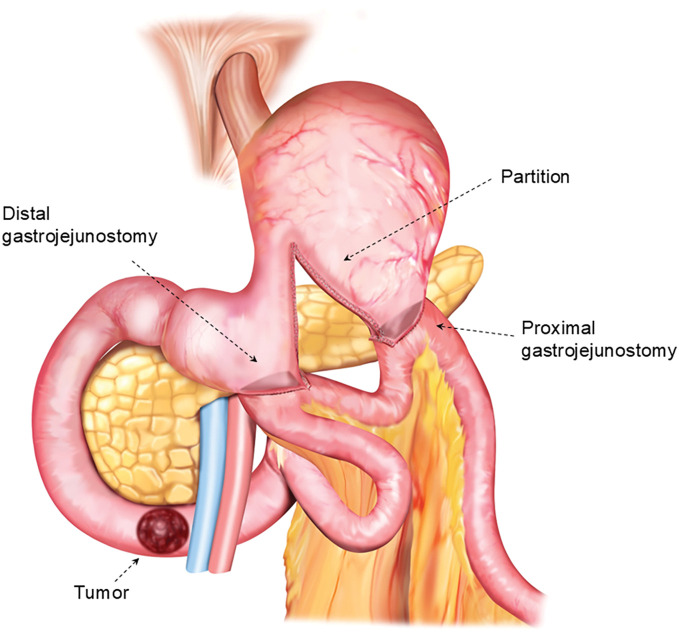
Schematic illustration of DO-SPGJ. The diagram shows the configuration of DO-SPGJ performed for malignant obstruction of the 3rd portion of the duodenum. A proximal gastrojejunostomy is created between the gastric body and a jejunal segment proximal to the obstruction, following incomplete gastric partitioning. An additional gastrojejunostomy is constructed between the gastric antrum distal to the partition and a more proximal jejunal loop, providing an outflow tract for digestive secretions. This dual-outlet design aims to prevent stagnation of bile and pancreatic juice and to maintain effective bypass function. DO-SPGJ, dual-outlet stomach-partitioning gastrojejunostomy

Subsequently, a conventional side-to-side SPGJ was performed between the gastric body proximal to the partition and a jejunal segment located approximately 10 cm distal to the previous anastomosis. For the proximal gastrojejunostomy, a 60-mm linear stapler was also used, and the cartridge was inserted nearly to its full length at both the gastric and jejunal walls. As a result, the actual length of the anastomosis was approximately 6 cm (**Fig. 2E**). The common enterotomy was temporarily closed with 3 stay sutures and then closed using a linear stapler (**Fig. 2F** and **2G**). All anastomoses were constructed via an antecolic route. An intraoperative view after completion of all anastomoses is shown in **Fig. 2H**. A schematic illustration of the completed DO-SPGJ is presented in **Fig. 3** to clarify the anatomical configuration of the 2 anastomoses. A drain was placed posterior to the anastomoses, and the procedure was completed. Operative time was 191 minutes with minimal blood loss (2 g).

### Postoperative course

**Fig. 4:**
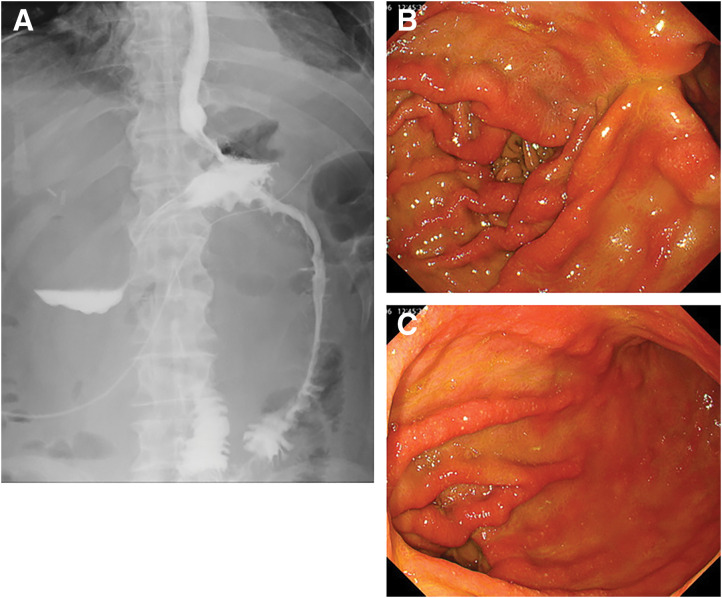
Postoperative contrast and endoscopic findings. (**A**) Upper gastrointestinal contrast study on POD 4 showed good passage through the gastrojejunostomy site without evidence of leakage. (**B**) Endoscopic view of the proximal gastrojejunostomy (oral side of the gastric partition) 1 month after surgery. (**C**) Endoscopic view of the distal gastric antrum and anastomosis (anal side of the gastric partition) 1 month after surgery.

On POD 4, an upper gastrointestinal contrast study with Gastrografin showed good passage through the anastomoses without leakage (**Fig. 4A**). Oral intake was resumed without difficulty, and the patient recovered without major complications.

Endoscopy at 1 month postoperatively confirmed the presence of 2 patent gastrojejunostomy sites with no evidence of stenosis (**Fig. 4B** and **4C**). Bile reflux into the stomach was observed, indicating functional bypass.

The patient resumed systemic chemotherapy without postoperative sequelae and maintained oral intake. At 3 months postoperatively, the patient maintained stable oral intake without nausea or vomiting. His body weight remained stable, suggesting preserved nutritional status postoperatively.

## DISCUSSION

We devised and implemented DO-SPGJ, a novel modification of SPGJ for malignant obstruction of the 3rd portion of the duodenum by adding a 2nd gastrojejunostomy to the distal side of the incomplete gastric partition. DO-SPGJ allowed drainage of digestive fluids from the duodenum and distal stomach, preventing their stagnation. The patient had an uneventful postoperative recovery with preserved oral intake. To our knowledge, this is the 1st report describing the use of DO-SPGJ in the setting of distal duodenal obstruction.

SPGJ has been reported to reduce delayed gastric emptying and tumor bleeding compared with conventional gastrojejunostomy.^4)^ Laparoscopic SPGJ is increasingly performed and has demonstrated safety and feasibility with faster resumption of oral intake compared with open approaches.^6)^ It is considered an effective palliative option for gastric outlet obstruction.^7)^

However, in cases where the obstruction lies distal to the ampulla of Vater, particularly in the 3rd portion of the duodenum or proximal jejunum, digestive secretions such as bile and pancreatic juice may become trapped. This can cause abdominal pain, vomiting, secondary cholangitis, or pancreatitis. The current SPGJ design may be insufficient to prevent this in certain cases. Although such events are not commonly reported after conventional SPGJ, similar issues have been documented following duodenal stent placement in the descending duodenum. Kaneko et al.^8)^ reported that biliary obstruction and pancreatitis occurred in a subset of patients due to compression or obstruction at the major duodenal papilla following self-expandable metal stent placement. Additionally, in chronic pancreatitis, concurrent biliary and duodenal obstruction has been associated with cholangitis and jaundice.^9)^

Although these scenarios differ from surgical gastrojejunostomy, they highlight the clinical relevance of digestive fluid stasis in the setting of distal duodenal obstruction. Therefore, our rationale for adding a 2nd, distal gastrojejunostomy was to ensure effective drainage of bile and pancreatic juice, thereby minimizing the risk of stasis-related complications. To our knowledge, no previous reports have described a similar dual-outlet reconstruction, and we believe this modification may offer a physiological advantage in selected patients.

The greatest strength of DO-SPGJ lies in its dual mechanism: it ensures effective bypass of the obstructed segment for ingested food via a proximal gastrojejunostomy, while simultaneously providing a distal outflow tract for bile and pancreatic secretions. The incomplete gastric partition plays a crucial role by preventing food from flowing into the distal stomach and duodenum, thereby minimizing the risk of reflux into the obstructed region. This configuration reduces the likelihood of digestive fluid stasis and related complications such as abdominal pain, vomiting, or cholangitis. As such, DO-SPGJ offers a more physiological and comprehensive solution compared with conventional bypass techniques, particularly in cases of distal duodenal obstruction.

Furthermore, by anatomically separating the routes for food and bile/pancreatic secretions, DO-SPGJ may help reduce the risk of afferent loop syndrome, which can occur due to stasis or pressure buildup in single-loop reconstructions. This structural separation may offer additional long-term benefits in select patients.

Few reports have addressed gastrojejunostomy for distal duodenal obstruction, but some case studies have described SPGJ for unresectable pancreatic or duodenal malignancies.^10,11)^

Several reports have described treatment methods for cases in which not only food but also bile and pancreatic juice stagnation becomes problematic. Mann et al.^12)^ evaluated the efficacy and safety of combined biliary and gastric bypass as palliative treatment for unresectable pancreatobiliary malignancies, and advocated its usefulness in patients expected to have a relatively long survival, given the procedure’s long-term stability. Sugerman et al.^13)^ also reported that, in cases of combined obstruction of the duodenum, pancreatic duct, and bile duct due to chronic pancreatitis, the addition of biliary drainage to gastrojejunostomy contributed to symptom improvement. These treatment methods are considered effective when the obstruction involves not only the duodenum but also the bile and pancreatic ducts. However, in cases where the obstruction is limited to the distal duodenum, adding biliary bypass or drainage is not essential and may be excessively invasive. While SPGJ, owing to its incomplete gastric partition, may prevent complete stagnation of bile and pancreatic juice within the duodenum, some degree of stasis in the gastric antrum or duodenum is still a concern due to the partitioning. We believe that our technique, which adds a 2nd gastrojejunostomy to the distal side of the partitioned stomach, may provide a solution to this issue.

The jejunal segment for the distal gastrojejunostomy was selected approximately 20 cm distal to the ligament of Treitz, consistent with common practice in duodenojejunostomy and gastrojejunostomy procedures, where 15–20 cm is frequently used as the standard distance.^14)^ This length allows for adequate reach, minimizes tension, and avoids mesenteric twisting.

The proximal gastrojejunostomy was constructed approximately 10 cm distal to the 1st anastomosis, creating sufficient space between the 2 anastomoses. This separation not only prevents overlapping of mesenteric vessels but also facilitates optimal angulation and ease of construction for both anastomoses.

In our case, the total operative time was 191 minutes with minimal blood loss. However, the patient had a history of multiple abdominal surgeries, and approximately 25 minutes were required for adhesiolysis before proceeding with the bypass. Therefore, the actual time required for the dual gastrojejunostomy was approximately 160 minutes.

Previous reports of laparoscopic gastrojejunostomy for malignant gastric outlet obstruction have reported mean operative times generally ranging from approximately 110 to 160 minutes, depending on factors such as the complexity of the case, prior surgeries, and whether additional procedures were performed.^7,15–17)^ While the operative time in our case was slightly longer than average, it is within a reasonable range considering the dual anastomoses required by DO-SPGJ and the technical demands of this novel reconstruction. Moreover, this was our 1st case of DO-SPGJ, and operative time may decrease as experience with the technique accumulates.

However, DO-SPGJ has some disadvantages. First, the procedure is more complex than conventional SPGJ due to the additional anastomosis. It also requires the use of more stapling devices, thereby increasing the cost. Furthermore, the stomach must be healthy enough to allow for 2 anastomoses over a wide area. Nevertheless, since DO-SPGJ is primarily indicated for distal duodenal obstruction, this requirement is typically met. Either stapling devices or barbed sutures may be used to close the common enterotomies. However, when using stapling devices, care should be taken to avoid anastomotic narrowing, and closure on the distal side—particularly when the anastomosis lies on the posterior gastric wall—may be technically challenging due to limited access and angulation. An additional advantage of DO-SPGJ is that it may also relieve food stagnation in the event that some ingested contents pass beyond the partition.

## CONCLUSIONS

We presented DO-SPGJ, a novel modification of stomach-partitioning gastrojejunostomy designed to address the challenge of digestive fluid stasis in patients with malignant obstruction of the 3rd portion of the duodenum. By incorporating a 2nd gastrojejunostomy distal to the gastric partition, this technique provides effective food passage while also facilitating physiologic drainage of biliary and pancreatic secretions. The procedure was safely performed laparoscopically and led to favorable postoperative outcomes. While further validation in additional cases is warranted, this approach may offer a promising option for selected patients with similar anatomical and pathological conditions.

## DECLARATIONS

### Availability of data and materials

The datasets supporting the conclusions of this article are included within the article.
